# Sexual behaviors and associated factors among youths in Nekemte town, East Wollega, Oromia, Ethiopia: A cross-sectional study

**DOI:** 10.1371/journal.pone.0220235

**Published:** 2019-07-29

**Authors:** Zelalem Desalegn Waktole

**Affiliations:** Department of Public Health, Institute of Health Science, Wollega University, Nekemte, Oromia, Ethiopia; University of Westminster, UNITED KINGDOM

## Abstract

**Background:**

Recent trends in sexual behavior, demonstrated in most countries, continue to indicate that more people are adopting safer sexual behaviors. However, there are signs of an increase in risky sexual behaviors in several countries. This study aimed to assess sexual behaviors and associated factors among youths in Nekemte town, East Wollega, Ethiopia in 2017.

**Methods:**

A community-based cross sectional study was conducted using a self-administered questionnaire. Then, the collected data were analysed using logistic regressions with 95% confidence interval (CI). Besides, the results of data analysis were `presented using appropriate descriptive measures and tables.

**Findings:**

Almost half of the respondents, 144(48.6%) had practiced sexual intercourse. Factors associated with ever had sexual intercourse include: being in age group 20-24(AOR = 2.322, 95% CI (1.258, 4.284)), having pocket money (AOR = 1.938, 95% CI (1.057, 3.556)), not attending school (AOR = 2.539, 95% CI (1.182, 5.456)), watching pornography (AOR = 4.314, 95% CI (2.265, 8.216)) and drinking alcohol (AOR = 7.725, 95% CI (3.077, 19.393)).

**Conclusion:**

High proportions of youths were practicing sexual intercourse. Targeting those identified associated factors in future intervention plan would improve the sexual behaviors of youths.

## Introduction

Youths are defined as those belonging to the age group of 15 to 24 years. Many important life events and health-damaging behaviors start during the youth years. As a result, youth is a time of both risk and opportunity. The period between the initiation of sexual activity and marriage is often a time of sexual experimentation and may involve risky behaviors [1, 2, 3].

HIV/AIDS has become one of the world’s most serious health and development challenges for the last three decades. UNAIDS reported that globally 33.3 million people were living with HIV at the end of 2009. Sub-Saharan Africa has only 13% of the world’s population but home to more than two-thirds (68%) of people living with HIV which bears an inordinate share of the burden and young people form a significant number in the population [4, 5, 6]. Young people are at the center of the global AIDS epidemic and the 1.7 billion young people worldwide; 5.4 million are estimated to be HIV positive. Young people aged 15–24, account for 41% of new HIV infections (Among those 15 and over) [4, 7].

One of the most severe consequences of HIV/AIDS is the loss of young adults in their most productive years and Ethiopia is one of the most affected countries in East Africa. The expression of the pandemic in the country is primarily young, with the majority of the victims aged 15–24 years [8, 9, 10].

Risky sexual behavior is defined as unprotected vaginal, oral or anal intercourse. Young people who initiate sexual intercourse at an early age face a higher risk of becoming pregnant or contracting an STI than young people who delay the initiation of sexual activity [3, 11]. The burden of risky sexual behaviors is reflected in economic and psychological costs in addition to sexually transmitted infections (ISTs) [12, 13].

Recent trends in sexual behavior, demonstrated in most countries, continue to indicate that more people are adopting safer sexual behaviors. However, there are signs of an increase in risky sexual behaviors in several countries. Recent evidence indicates a significant increase in the number of sexual partners and a decline in condom use in some countries including Ethiopia [14].

Condom use during sexual intercourse is an effective method for avoiding pregnancy and infection from STIs [2]; however, a study showed that significant proportions of respondents do not always use a condom with non-regular partners, though they know that condom use protects from HIV infection. Some even thought that condom is less effective and /or potentially dangerous to disseminate HIV [15].

It is clear that youths living in an urban area are at higher risk because of their sexual behaviors [2]. Accordingly, this study tried to identify factors associated with the sexual behaviors of youths in Nekemte town and the results would contribute as an input for a future intervention plan.

## Methods and materials

The study was conducted in Nekemte town, which is the capital city of East Wollega Zone, Oromiya National Regional State. Nekemte town is located 331 km away from the capital city of the country (Addis Ababa), in the Western direction. The town has 97,877 total populations and divided into six sub-cities. The estimated number of youths in the town with age group 15–24 is 22,480. The study was conducted from July 24 to 29, 2017. Community based quantitative cross-sectional study design was used to collect data using a self-administered questioner.

The sample size was calculated using a single population proportion formula by taking 21.5% prevalence of premarital sexual intercourse [16], 5% margin of error and confidence interval of 95%. A design effect of 2 was considered and yielded a 298 final sample size by adding 15% for non-response. Those who had been living in the town at least for the last six months before data collection dates were included.

All households which are found in each sub-city were initially mapped and numbered. The study subjects were recruited using probability proportional to the number of households in all sub-cities. Every Kth (the proportion of total youths in each sub-city divided by their respective sample size) of pre-numbered households were visited, until the required number of youths was identified using systematic random sampling. The direction of the initial household was determined by a spinning pen at the center of each sub-city. In case, when more than one eligible study participant was present in a given household, one of them was selected by simple random sampling. When the study participant was not found at home during data collection, an appointment was arranged to go back for data collection. If there were no eligible youth in the selected household, the next household was recruited.

The questionnaire was taken from similar studies and adapted to the context of this study [17–20]. It was self-administered and consists of background information, sexual experiences and non-sexual behaviors. Six data collectors, grade 10 completed and above were selected and recruited for data collection. One day training was given for data collectors. The training was based on the guide that was developed for data collection and clarifying self-administered questionnaires. Besides, they were trained on the overall procedures of data collection. The questionnaire was prepared in English and translated to Afan Oromo to make it clear for the participants and reduce misunderstanding. Respondents were told to complete the questioners and data collectors clarify any doubts raised during filling the questioners. The principal investigator supervised all activities.

To assure quality of the data properly designed data collection instrument was prepared and pre-tested for its clarity. The questionnaire was pre-tested among youths visiting the Family Guidance Association of Ethiopia Nekemte branch and excluded from the actual study. Based on feedback from the pre-test modifications were made. Data were checked for completeness and responses for each question were coded and entered to Statistical Package for Social Sciences (SPSS) version 20.0 by the principal investigator.

Then, the collected data were analysed using logistic regressions with 95% confidence interval (CI). Variables that were statistically significant in bivariate analysis were entered to multivariate analyses to identify the associated factors where the entry value was 0.05. Besides, the results of data analysis were `presented using appropriate descriptive measures and tables.

### Operational definitions

**Sexual behavior**: condom use during sexual intercourse, number of sexual partners, age of starting sexual intercourse and sexual intercourse with commercial sex workers.

**Risky sexual behavior**- Youth who have at least one of the following: inconsistent condom use during each sexual intercourse, having multiple sexual partners, starting sexual intercourse before age of 18 years and sexual intercourse with commercial sex workers.

**Sexual partner-** Girl/boyfriend or any person with whom sexual intercourse is practiced by the youths

**Regular sexual partner**- spouse who have sexual contact with the youth for more than 12 months

**Consistent condom use-**using condom appropriately during or at every sexual encounter

**Practiced HIV prevention methods:** respondent who ever practiced the major HIV/AIDS prevention methods (abstinence, being faithful to one partner or condom use)

**Religious participation:** respondent who have a religion and active member of any religious organization

### Ethics statement

The ethical approval letter was obtained from Wollega University ethical approval committee. A formal letter was written by Wollega University to each sub-city for their cooperation during data collection. Written consent was obtained from each study participants with 18 years old and above and for those under 18 years, oral consent was obtained from parents or guardians.

## Results

### Socio-demographic characteristics

Two hundred ninety-six (296) youths were participated in this study making a response rate of 99.33%. A total of 196 (66.2%) males and 100 (33.8%) females participated in this study. The mean age of the respondents was 19.70(+2.406). 141(47.6%) of the respondents were in the age group of 15–19 and 155(52.4%) were between 20–24 age group. 224(75.7%) of the respondents had religious participation while 72(24.3%) had not. Regarding the ethnicity of the respondents, 243(82.1%) were Oromo, 22(7.4%) Amhara, 13(4.4%) Tigre and 18(6.1%) were Gurage. Besides, 149(50.3%) had pocket money and 225(76.0%) of the youths had been attending school (Table 1).

**Table 1:** Socio-demographic characteristics of youths, Nekemte, Oromia, Ethiopia, 2017 (n = 296).

**Variables**	**Frequency (n)**	**Percent (%)**
Sex		
Male	196	66.2
Female	100	33.8
Age group		
15–19	141	47.6
20–24	155	52.4
Sub-city		
Kaso	54	18.2
Darge	47	15.9
Chalalaki	52	17.6
Kase	47	15.9
Baka Jama	51	17.2
Burka Jato	45	15.2
Religious participation		
Yes	224	75.7
No	72	24.3
Ethnicity		
Oromo	243	82.1
Amhara	22	7.4
Tigre	13	4.4
Gurage	18	6.1
Pocket money		
Yes	149	50.3
No	147	49.7
Attending school		
Yes	225	76.0
No	71	24.0

### Sexual and non-sexual behaviors of the respondents

Almost half of the respondents, 144(48.6%) ever had sexual intercourse. The majority, 102(70.8%) had their first sexual intercourse before 18 years with a mean age of 17.07(2.649). Out of those sexually active respondents, 73(50.7%) had regular sexual partners, 38(26.4%) had an occasional sexual partner and 33(22.9%) had no sexual partner during the study period. 30(20.8%) of them had sexual intercourse with commercial sex workers.

Besides, one hundred twelve (77.8%) of those sexually active respondents had practiced sexual intercourse in the last 12 months before data collection. 56(38.9%) had one sexual partner and 88(61.1%) had two or more sexual partners during the data collection period. 51(35.4%) of them had never used a condom, 32(22.2%) used sometimes, 18(12.5%) used most of the times and 43(29.9%) used always during the last 12 months before data collection. On the first sexual intercourse, 59(41%) had used a condom. The reasons mentioned for not using condom was not available 12(11.7%), dislike it 12(11.7%), partner refused 10(9.7%), trust on partner 18(17.5%), condom reduce pleasure 24(23.3%), I didn`t think of it 12(11.7%), condom may have virus 1(1%), condom is expensive 3(2.9%), in love with partner 7(6.8%) and others 4(3.9%).

On their last sexual intercourse, 76(52.8%) had used a condom. 57(39.6%) of the respondents had received money/gift to have sexual intercourse. Out of the total respondents, 158(53.4%) had viewed pornography, 73(24.7%) drink alcohol, 45(15.2%) chew khat and 22(7.4%) smoke cigarettes. The majority, 174(58.8%) had practiced major HIV/AIDS prevention methods and most of them, 207(69.9%) had checked serostatus (Tables 2 and 3).

**Table 2:** Sexual and non- sexual behaviors of youths at Nekemte town, Oromia, Ethiopia, 2017 (n = 296).

**Variables**	**Frequency (n)**	**Percent (%)**
Ever had sexual intercourse		
Yes	144	48.6
No	152	51.4
Watch pornography		
Yes	158	53.4
No	138	46.6
Drink alcohol		
Yes	73	24.7
No	223	75.3
Chew khat		
Yes	45	15.2
No	251	84.8
Smoke cigarette		
Yes	22	7.4
No	274	92.6
Practice HIV prevention methods		
Yes	174	58.8
No	122	41.2
Checked serostatus		
Yes	207	69.9
No	89	30.1

**Table 3:** Sexual behaviors of youths who had sexual intercourse at Nekemte town, Oromia, Ethiopia, 2017 (n = 144).

**Variables**	**Frequency (n)**	**Percent (%)**
Age at first sexual intercourse		
<18	102	70.8
≥18	42	29.2
Do you have sexual partner?		
Yes, regular	73	50.7
Yes, occasional	38	26.4
No	33	22.9
Sexual intercourse with commercial sex workers		
Yes	30	20.8
No	114	79.2
Last 12 months sexual intercourse		
Yes	112	77.8
No	32	22.2
Number of sexual partner		
1	56	38.9
≥2	88	61.1
Last 12 months condom use		
Never used	51	35.4
Sometimes	32	22.2
Most of the times	18	12.5
Always	43	29.9
First sexual intercourse condom use		
Yes	59	41
No	85	59
Reasons for not using condom		
Condom not available	12	11.7
I dislike it	12	11.7
My partner refused	10	9.7
I have trust on my partner	18	17.5
Condom reduces pleasure	24	23.3
I did not think of it	12	11.7
Condom may have virus	1	1
Condom is expensive	3	2.9
I am in love with my partner	7	6.8
Others*	4	3.9
Last sexual intercourse condom use		
Yes	76	52.8
No	68	47.2
Exchanged money or gift for sexual intercourse		
Yes	57	39.6
No	87	60.4

*virgin sexual intercourse doesn’t need condom use

## Discussion

This study tried to identify the sexual behaviors of youths in Nekemte town. Almost half of the respondents were ever had sexual intercourse, which indicates that youths are in high risk and need special attention. This result is similar to another similar study conducted in Dilla town [17]. However, it is higher than studies conducted on Ugandan university students and university students in Ethiopia [18, 19]. This difference might be related to the characteristics of the study population and study area. However, it is lower than studies conducted in Yabello and Addis Ababa [20, 21]. The majority of those who ever had sexual intercourse started their first sexual intercourse before 18 years, which exposed them to more risks. This result is consistent with other similar studies [17, 20]. However, it is lower than the study results from Madawalabu University [22].

Out of those sexually active respondents, only half of them had a regular sexual partner. This means, half of them committed sexual intercourse with different people, which is another indicator that youths are in unsafe sexual behavior. This study also revealed that a significant number of respondents had sexual intercourse with commercial sex workers which are higher than a study conducted in Dilla town [17]. This result shows that youths are practicing risky sexual behaviors that expose them to sexually transmitted diseases. However, it is lower than in other studies [19, 22].

The majority of those sexually active respondents, 77.8% had practiced sexual intercourse the last 12 months before data collection. This is higher when compared with the previous study conducted in the same area [23]. This is also another alarming result for those who are working on this issue. Sticking to one sexual partner for those sexually active is better to reduce risks; however, this study revealed that the majority of the respondents had two or more sexual partners during the data collection period. This is higher when compared to results from other studies [17– 20, 22–23]. This difference might be due to the characteristics of the study participants and the study area.

Only less than one third among sexually active respondents had used condom consistently in the last 12 months before data collection. The main reasons mentioned for not using a condom were unavailability, dislike it, partner refused and condom reduces pleasure. The results are consistent with other similar studies [17, 19, 22, 24]. Consistent condom use is one of the major HIV/AIDS prevention methods. Those different reasons identified for not using a condom by this study would contribute to promoting consistent condom use.

Condom use on the last sexual intercourse was higher than the first sexual intercourse; this could be related to the fact that most of the first sexual intercourse is accidental. More than two-thirds had exchanged money or gift to commit sexual intercourse. This result is similar to other studies [17, 19, 24]. The reason could be related to that youths are attracted by silly materials. More than half of the respondents in this study had watched pornography. This might be related to easily accessible media and the internet. Concerning substance use, drink alcohol, chew khat and cigarette smoking were identified in order of its magnitude by this study.

Youths with age group 20–24 were more likely had sexual intercourse when compared to 15–19 age groups (AOR = 2.322; 95% CI: 1.258, 4.284). The difference might be due to less family supervision as age advanced. Those who had pocket money were more likely committed sexual intercourse than those who did not have pocket money (AOR = 1.938; 95% CI: 1.057, 3.556). The reasons could be related to the mishandling of money. Youths who were not attending school were more likely committed sexual intercourse than those who were attending school (AOR = 2.539; 95% CI: 1.182, 5.456). This difference might be due to more exposure of the youths to factors like peer pressure.

Those who watched pornography were more likely committed sexual intercourse than those who did not watch pornography (AOR = 4.314; 95% CI: 2.265, 8.216). This might be due to the motivation that the youths develop from viewing pornography to exercise sexual intercourse. Those who drink alcohol were more likely committed sexual intercourse when compared with those who did not drink (AOR = 7.725; 95% CI: 3.077, 19.393). This might be related to fail to self-control and commit unplanned sexual intercourse. Those who did not practice major HIV/AIDS prevention methods were less likely committed sexual intercourse when compared with their counterparts (AOR = 0.243; 95% CI: 0.128, 0.460) (Table 4).

**Table 4:** Factors associated to ever had sexual intercourse with independent variables among youths in Nekemte, Oromia, Ethiopia, 2017 (n = 296).

Ever had sexual intercourse				
Variables	Yes (%) (n = 144)	No (%) (n = 152)	COR (95% CI)	AOR (95% CI)
Age group				
15–19	45(31.2)	96(63.2)	1.00	1.00
20–24	99(68.8)	56(36.8)	3.771 (2.328–6.110)*	2.322(1.258–4.284)**
Religious participation				
Yes	97(67.4)	127(83.6)	1.00	1.00
No	47(32.6)	25(16.4)	2.461(1.417–4.277)*	1.498 (0.732–3.065)
Have pocket money				
Yes	95(66.0)	54(35.5)	3.519 (2.180–5.679)*	1.938 (1.057–3.556) **
No	49(34.0)	98(64.5)	1.00	1.00
Attending school				
Yes	97(67.4)	128(84.2)	1.00	1.00
No	47(32.6)	24(15.8)	2.584(1.479–4.515)*	2.539(1.182–5.456) **
Watch pornography				
Yes	105(72.9)	53(34.9)	5.029 (3.061–8.261)*	4.314(2.265–8.216) **
No	39(27.1)	99(65.1)	1.00	1.00
Drink alcohol				
Yes	62(43.1)	11(7.2)	9.692(4.829–19.451)*	7.725(3.077–19.393) **
No	82(56.9)	141(92.8)	1.00	1.00
Chew khat				
Yes	34(23.6)	11(7.2)	3.962(1.921–8.173)*	0.568(0.173–1.868)
No	110(76.4)	141(92.8)	1.00	1.00
Smoke cigarette				
Yes	18(12.5)	4(2.6)	5.286(1.743–16.025)*	0.957(0.178–5.138)
No	126(87.5)	148(97.4)	1.00	1.00
Practice HIV prevention methods				
Yes	106(73.6)	68(44.7)	1.00	1.00
No	38(26.4)	84(55.3)	0.290 (0.178–0.473)*	0.243(0.128–0.460)**
Checked serostatus				
Yes	109(75.7)	98(64.5)	1.00	1.00
No	35(24.3)	54(35.5)	0.583 (0.352–0.966)*	0 .855(0.427–1.711)

*significant for crude OR

**significant for Adjusted OR

## Conclusions

Almost half of the respondents were sexually active and the majority had their first sexual intercourse before 18 years. A significant number of youths had sexual intercourse with commercial sex workers. About two third of them had two or more sexual partners during the data collection period. Only less than one third used condom consistently during the last 12 months before data collection.

Condom use of last sexual intercourse was slightly higher than first sexual intercourse but not satisfactory. There were misconceptions and other reasons identified for not using a condom that needs interventions. Being in the age group 20–24, having pocket money, not attending school, watching pornography and drinking alcohol were identified associated factors with ever had sexual intercourse.

Focusing on those identified variables in future intervention plan would improve the sexual behaviors of youths in the town. Production and distribution of IEC (Information, Education, and Communication) materials to avoid misconceptions of youths on condom use should be enhanced. Supporting peer education on HIV/AIDS prevention and consequences of teenage sexual debut would improve the sexual behavior of youths.

## Supporting information

S1 Appendix Study Questionnaire in English and Afan Oromo. (PDF)

S1 Dataset Questionnaire Results Data in SPSS Format. (SAV)
