# Supplementary material for: Sexual behaviors and associated factors among youths in Nekemte town, East Wollega, Oromia, Ethiopia: A cross-sectional study
Source: PLoS One. 2019 Jul 29;14(7):e0220235. doi: 10.1371/journal.pone.0220235 (PMC6663068; doi:10.1371/journal.pone.0220235)
Supplement: S1 Appendix — (PDF) [file pone.0220235.s001.pdf]

Annex-I Questioner ‘English’

Hello, how are you? My name is ___________________________. I am a member of data collectors on “Sexual behaviors and associated factors among youth in Nekemte town”.

The objective of this study is to identify factors associated with Sexual behaviors for intervention.

In case, if you have any question and doubts you can ask me.

Thankyou for your cooperation!

Consent

Are you voluntary to give us information? Yes _____ No_____

Name of data collector _____________________ Signature ______________________ Date ________________

Section- 1 Identification and Background

No	Questions	Coding category	Skip to
0	Kebele	______________________	
1	Sex	Male----------------------------1<br>Female--------------------------2	
2	Your age in years	_______	
3	Do you have religious participation?	Yes----------------------------------1<br>No------------------------------------0	
4	What is your ethnicity?	Amhara-------------------------1<br>Oromo---------------------------2<br>Tigre----------------------------3<br>Gurage---------------------------4<br>Others----------------------------8<br>(Specify)_________________9	
5	Do you have Pocket Money	Yes----------------------1<br>No-----------------------0	
6	Are you currently attending school?	Yes----------------------1<br>No-----------------------0	

Section-2 Behaviours

No	Question & filter	Coding category	Skip to
1	Have you ever had sexual intercourse?	Yes-----------------------1<br>No-----------------------0 _____→	 12
2	How old were you when you started Sexual intercourse?	Age in years_____	
3	Do you currently have sexual partner?	No, --------------------------------0<br>Yes, regular sexual partner------2<br>Yes, occasional sexual partner—3	
4	Have you ever had sexual intercourse with commercial sex workers?	Yes----------------------------------1<br>No-----------------------------------0	
5	Have you had sexual intercourse during the last 12 months?	Yes----------------------------------1<br>No-----------------------------------0	
6	How many different sexual partners do you have in your lifetime?	Number_________<br>I can't remember____	
7	How frequently were you using condom in the last 12 months?	Never used----------------------------1<br>Sometimes----------------------------2<br>Most of the times--------------------3<br>Always--------------------------------4	
8	The first time you have sex with your partner did you use condom to prevent HIV infection?	Yes----------------------------------1<br>No-----------------------------------0	
9	If you were not using condom every time what was /were the reasons	Condom not available---------------1<br>I dislike it----------------------------2<br>My partner refused------------------3<br>I have trust on my partner----------4<br>Condoms reduce pleasure----------5<br>I didn`t think of it--------------------6<br>Condoms may have virus----------7<br>Condoms are expensive------------8<br>I am in love with my partner------9<br>Others--------------------------------10<br>(please specify)_________________	
10	The last time you had sex with your partner did you use condom to prevent HIV infection	Yes----------------------------------1<br>No-----------------------------------0	
11	Received money/gift to have sex for past 12 month	Yes----------------------------------1<br>No-----------------------------------0	
12	Ever Viewed pornographic Films	Yes----------------------------------1<br>No-----------------------------------0	

13	Currently do you drink alcohol?	Yes-----------------------------------1<br>No------------------------------------0	
14	Currently do you chew Khat?	Yes-----------------------------------1<br>No------------------------------------0	
15	Currently do you smoke Cigarette?	Yes-----------------------------------1<br>No------------------------------------0	
16	Have you ever practiced the major HIV prevention methods?	Yes-----------------------------------1<br>No------------------------------------0	
17	Have you ever checked sero-status?	Yes-----------------------------------1<br>No------------------------------------0	
18	Have you ever visited FGA?	Yes-----------------------------------1<br>No------------------------------------0	
19	What services have been offered by FGA?	_______________	

Anex-II Questioner ‘Afan Oromo’

Kaayyoon qorannoo kanaa amala saal-qunnamtii dargaggootaa magaalaa Naqamtee adda baasuun tajaajila barbaachisaa ta’e akka argatan taasisa. Gaaffii fi yaada ifa siif hin taane yoo qabaatte sodaa tokko malee nagaafadhu.

Unka walii galtee

Odeeffannoo gabaabaa qorannoo kanaaf oolu naaf kennuuf fedha qabdaa? Eeyyee Lakki

Maqaa nama odeeffannoo funaanee Mallattoo Guyyaa

____________________ ____________________ ________________

Kutaa- 1 Gaaffilee eenyummaa fi seenaa

Lakk.	**Gaaffilee**	Koodii	
0	Ganda	______________________	
1	Saala	Dhiira ----------------------------1<br>Dhalaa ---------------------------2	
2	Umurii	_______	
3	Amantaakee keessatti hirmaannaa qabdaa?	Eeyyee ---------------------------1<br>Lakki ------------------------------0	
4	Qomoonkee maali?	Oromoo --------------------------1<br>Amaara ---------------------------2<br>Tigiree-----------------------------3<br>Guragee---------------------------4<br>kanbiroo--------------------------8<br>(barreessi)___________-____9	
5	Maallaqa guyya guyyaatti itti fayyadamtu ni argattaa?	Eeyyee -----------------------------1<br>Lakki -------------------------------0	
6	Barumsa barachaa jirtaa?	Eeyyee -----------------------------1<br>Lakki -------------------------------0	

kutaa-2 Amaloota wal-qunnamtii saalaa waliin wal-qabatan

Lak.	Gaaffilee	Koodii	
1	Wal-qunnamtii saalaa gootee beektaa?	Eeyyee --------------------------1<br>Lakki ----------------------------0	Lakkii gaaffii 12 darbi
2	Yeroo wal-qunnamtii saalaa jalqabdu umuriinkee meeqa ture?	_____________	
3	Hiriyyaa wal-qunnamtii saalaa waliin raawwattu qabdaa?	lakki, -----------------------------------0<br>Eeyyee, hiriyaa dhaabbataa-----------1<br>Eeyyee, kan dhaabbataa hin taane--2	
4	Warra mana bunaa hojjetan waliin wal-qunnamtii saalaa raawwattee beektaa?	Eeyyee --------------------------1<br>Lakki ----------------------------0	
5	Ji'a 12 darbe keessa wal-qunnamtii saalaa raawwatteettaa?	Eeyyee --------------------------1<br>Lakki ----------------------------0	
6	Hanga harraatti hiriyaa meeqa waliin wal-qunnamtii saalaa raawwatte?	_________<br>Hin yaadadhu ____	
7	Ji'a 12 darbe keessa yeroo meeqa kondomii fayyadamte?	Hin fayyadamne---------------------1<br>Darbee darbee------------------------2<br>Harka caalu---------------------------3<br>Yeroo hundumaa--------------------4	
8	Yeroo jalqaba wal-qunnamtii saalaa raawwatte kondomii fayyadamteettaa?	Eeyyee -------------------------------1<br>Lakki ---------------------------------0	
9	Koondomii fayyadamaa hin turre yoo ta'e sababnisaa maali?	Koondomii hin arganne-----------1<br>Hin jaalladhu------------------------2<br>Hiriyaakootu dide-------------------3<br>Hiriyaakoo waanan amanuuf-----4<br>Kondomiin gammachuu hirrisa --5<br>Itti hin yaadne -----------------------6<br>Kondomiin vaayirasii qabaachuu danda'a---------------------------------7<br>Gatiin koondomii miyaadha -------8<br>Hiriyaakoo waanan jaalladhuuf----9<br>kanbiroo-------------------------------10<br>(barreessi)________________	
10	Wal-qunnamtii saalaa dhuma kana raawwatte irratti koondomii fayyadamtee?	Eeyyee ----------------------------1<br>Lakki ------------------------------0	

11	Ji’a 12n darbe keessa wal-qunnamtii saalaa raawwachuuf maallaqa ykn kennaa fudhatteettaa/laatteettaa?	Eeyyee ----------------------------1<br>Lakki ------------------------------0	
12	Fiilmii wal-qunnamtii saalaa qabu ni daawwattaa?	Eeyyee ----------------------------1<br>Lakki ------------------------------0	
13	Alkoolii ni dhugdaa?	Eeyyee ----------------------------1<br>Lakki ------------------------------0	
14	Caatii/Jimaa ni qamaataa?	Eeyyee ----------------------------1<br>Lakki ------------------------------0	
15	Tamboo ni xuuxxaa?	Eeyyee ----------------------------1<br>Lakki ------------------------------0	
16	Maloota HIV ittiin ittisan fayyadamtee beektaa? (Of qusachuu, amanamuu, kondomii fayyadamuu)	Eeyyee ----------------------------1<br>Lakki ------------------------------0	
17	HIV dhaaf dhiigakee qoratamtee beektaa?	Eeyyee ----------------------------1<br>Lakki ------------------------------0	
18	Dhaabbata Waldaa Qajeelcha Maatii Damee Naqamtee deemtee beektaa? Tajaajila argachuuf ykn bashannanuuf	Eeyyee ----------------------------1<br>Lakki ------------------------------0	
19	Tajaajiloota dargaggoonni achii argatan maalfaadha? Barreessi	______________________________	
